# Urinary proteomics identifies distinct immunological profiles of sepsis associated AKI sub-phenotypes

**DOI:** 10.1186/s13054-024-05202-9

**Published:** 2024-12-18

**Authors:** Ian B. Stanaway, Eric D. Morrell, F. Linzee Mabrey, Neha A. Sathe, Zoie Bailey, Sarah Speckmaier, Jordan Lo, Leila R. Zelnick, Jonathan Himmelfarb, Carmen Mikacenic, Laura Evans, Mark M. Wurfel, Pavan K. Bhatraju

**Affiliations:** 1 Division of Nephrology, Department of Medicine, Kidney Research Institute, University of Washington Medical Center, 325 9th Avenue, Seattle, WA 98104 USA; 2 Sepsis Center of Research Excellence, SCORE-UW), University of Washington, Seattle, WA USA; 3 Division of Pulmonary, Critical Care and Sleep Medicine, Department of Medicine, University of Washington Medical Center, Seattle, WA USA; 4 Translational Research, Benaroya Research Institute, Seattle, WA USA

**Keywords:** Urinary proteomics, Sepsis, Intensive care unit, Acute kidney injury

## Abstract

**Background:**

Patients with sepsis-induced AKI can be classified into two distinct sub-phenotypes (AKI-SP1, AKI-SP2) that differ in clinical outcomes and response to treatment. The biologic mechanisms underlying these sub-phenotypes remains unknown. Our objective was to understand the underlying biology that differentiates AKI sub-phenotypes and associations with kidney outcomes.

**Methods:**

We prospectively enrolled 173 ICU patients with sepsis from a suspected respiratory infection (87 without AKI and 86 with AKI on enrollment). Among the AKI patients, 66 were classified as AKI-SP1 and 20 as AKI-SP2 using a three-plasma biomarker classifier. Aptamer-based proteomics assessed 5,212 proteins in urine collected on ICU admission. We compared urinary protein abundances between AKI sub-phenotypes, conducted pathway analyses, tested associations with risk of RRT and blood bacteremia, and predicted AKI-SP2 class membership using LASSO.

**Measurement and main results:**

In total, 117 urine proteins were higher in AKI-SP2, 195 were higher in AKI-SP1 (FDR < 0.05). Urinary proteins involved in inflammation and chemoattractant of neutrophils and monocytes (CXCL1 and REG3A) and oxidative stress (SOD2) were abundant in AKI-SP2, while proteins involved in collagen deposition (GP6), podocyte derived (SPOCK2), proliferation of mesenchymal cells (IL11RA), anti-inflammatory (IL10RB and TREM2) were abundant in AKI-SP1. Pathways related to immune response, complement activation and chemokine signaling were upregulated in AKI-SP2 and pathways of cell adhesion were upregulated in AKI-SP1. Overlap was present between urinary proteins that differentiated AKI sub-phenotypes and proteins that differentiated risk of RRT during hospitalization. Variable correlation was found between top aptamers and ELISA based protein assays. A LASSO derived urinary proteomic model to classify AKI-SP2 had a mean AUC of 0.86 (95% CI: 0.69–0.99).

**Conclusion:**

Our findings suggest AKI-SP1 is characterized by a reparative, regenerative phenotype and AKI-SP2 is characterized as an immune and inflammatory phenotype associated with blood bacteremia. We identified shared biology between AKI sub-phenotypes and eventual risk of RRT highlighting potential therapeutic targets. Urine proteomics may be used to non-invasively classify SP2 participants.

**Supplementary Information:**

The online version contains supplementary material available at 10.1186/s13054-024-05202-9.

## Introduction

Acute kidney injury (AKI) is the most common form of organ failure in sepsis [1]. Persons who develop sepsis-induced AKI are at greater risk of inpatient need for renal replacement therapy and future chronic kidney disease (CKD) and end-stage renal kidney disease (ESKD)) [2–7]. The mechanisms underlying AKI in sepsis are diverse, involving macrovascular and microvascular dysfunction, inflammatory injury leading to tubular cell dysfunction, cellular apoptosis, cell-cycle arrest and others [8, 9]. Despite the substantial clinical impact of sepsis-induced AKI, multiple clinical trials have yet to identify effective pharmacotherapy for its prevention or treatment [5, 10]. One reason for the lack of therapeutics may be the presence of biologically distinct AKI subtypes that conceal identification of therapeutic targets to prevent and treat sepsis-induced AKI in clinical populations [11–17].

Traditional models of pharmacotherapy in AKI have focused on developing therapies that could be provided to broad AKI populations. In contrast, an AKI sub-phenotype model presumes the existence of physiologic subtypes that require different therapies that target distinct disease pathways [18]. Our group and others have identified two distinct AKI sub-phenotypes (AKI-SP1 and AKI-SP2). To ease clinical identification of these AKI sub-phenotypes, we developed and validated a 3-variable model that included plasma markers of endothelial dysfunction (angiopoietin-1 (Ang-1) and angiopoietin-2 (Ang-2)) and inflammation (soluble tumor necrosis factor receptor-1 (sTNFR-1)). These AKI sub-phenotypes demonstrated different risk of short and long-term clinical outcomes and also had distinct genetic risk [19–21]. We also leveraged these AKI sub-phenotypes to demonstrate that existing therapies in sepsis (early addition of vasopressin) may preferentially lead to renal recovery in one AKI sub-phenotype (AKI-SP1) compared to the other (AKI-SP2). However, to identify new therapies tailored to AKI sub-phenotypes requires a deeper understanding of the biological pathways characterizing each AKI sub-phenotype.

In this study, we leverage urine sampling on ICU admission paired with detailed clinical phenotyping. Urine is an ideal biofluid for the study of kidney diseases because it can be collected non-invasively and a majority of the urinary proteome derives from the kidney [22]. We use the Somascan aptamer platform to measure ~ 5000 proteins to understand key reparative, inflammatory and fibrotic pathways underlying AKI sub-phenotypes. The following analytical steps were completed: first, AKI sub-phenotypes were defined using a 3 variable plasma prediction model. Second, urine proteomics were compared between AKI sub-phenotypes. Third, pathway analyses were completed to understand distinct biological processes involved in AKI sub-phenotypes. Fourth associations with urinary proteins and risk of RRT were tested to identify shared biology between AKI sub-phenotypes and kidney related clinical outcomes. Finally, we developed a urinary proteomic prediction model using Least Absolute Shrinkage and Selection Operator (LASSO) to classify AKI sub-phenotypes and overcome the need for blood sampling.

## Methods

### Study population

We conducted a prospective cohort study of critically ill patients admitted to three hospitals affiliated with the University of Washington (Seattle, WA). Patients were enrolled between March 2020 and May 2021 [23–25]. Patients were eligible if admitted to an ICU with signs or symptoms of acute respiratory infection (fever, respiratory symptoms including cough/shortness of breath or sore throat) and had one of the following 1) initiation of supplemental oxygen; 2) oxygen saturation < 94% on ambient air; or 3) new opacities on chest radiograph. We excluded patients who were younger than 18 years, incarcerated, pregnant, or on chronic maintenance hemodialysis. For this ancillary study, we selected all enrolled participants who had available spot urine and blood samples within 24 h of ICU admission. All urine samples were collected from the sampling port from an indwelling urinary catheter and all urine and blood samples were collected within 2 h of each other. Collecting urine from the sampling port ensured recently produced urine. Urine was immediately centrifuged and aliquoted within 2 h of collection. All urine samples were then frozen and underwent a single-freeze thaw for urine proteomics.

The University of Washington Human Subjects Division granted a waiver of informed consent given minimal risk, urgency of COVID-19 research in this period, and supply limitations in personal protective equipment preventing nonessential staff from approaching patients (STUDY #9763).

### Sample collection, proteomic platform, and quality control

Peripheral blood was collected into EDTA anticoagulant tubes within 24 h of ICU admission. Plasma was isolated by centrifugation (10 min, 2000* g*, room temperature). We measured Ang-1, Ang-2 and sTNFR-1 using electrochemiluminiscence-based immunoassays (Meso Scale Discovery, Rockville, MD). Biomarkers were measured in 2 batches and the inter-plate coefficient of variation for Ang-1 was 6.2%, Ang-2 was 10.3% and sTNFR-1 was 7.3%. All samples underwent 1 freeze–thaw cycle prior to analysis.

Urine proteomic profiling was completed using the SomaScan Platform (Somalogic) that contains SOMAmer single-stranded DNA aptamers that bind to protein analytes with high specificity. The assays were performed as previously described [26–28]. For each sample, the platform reported a relative fluorescence units (RFU) value for each aptamer-protein pair that provides a scale-free measure of protein abundance. Median intra- and inter-assay coefficients of variation are approximately 5% [29]. Samples were analyzed in two batches, on the Somalogic v4 platform with 5212 aptamers that bind 4925 unique proteins, and the v4.1 platform with 7548 aptamers that bind 6399 unique proteins. Analyses were conducted on the set of 5212 aptamers that overlapped between the v4 and v4.1 Somalogic platforms.

### AKI and AKI sub-phenotype identification

AKI was defined as an increase in serum creatinine (SCr) at the time of study enrollment of ≥ 0.3 mg/dl or 50% from a baseline SCr consistent with the KDIGO guidelines [30]. Patients receiving dialysis prior to study enrollment were excluded. Among the patients without AKI on study enrollment, a subset subsequently developed AKI during hospitalization. However, since our primary analysis was to link differences in urinary protein concentrations with AKI sub-phenotypes, we did not include patients who developed AKI after study enrollment to classify AKI sub-phenotypes. The outcome of renal replacement therapy (RRT) was defined as new initiation of RRT during hospitalization.

The baseline SCr was a pre-hospitalization SCr within 365 days and if a pre-hospitalization SCr was missing then the baseline was the lowest SCr value within 7 days of study enrollment. Among patients with AKI at study enrollment, we used plasma biomarker concentrations of Ang-1, Ang-2 and sTNFR-1 to identify two AKI sub-phenotypes (AKI-SP1 and AKI-SP2). The model to identify AKI sub-phenotypes is a previously reported 3-variable prediction model, *Logit (P(AKI sub-phenotype membership))* = *-41.246* + 5.241*log(Ang-2/Ang-1) + 3.242*log(TNFR-1). A Youden’s index cutoff of 0.403 was used with greater values classifying patients as AKI-SP2 and values less than 0.403 classifying patients as AKI-SP1 [19].

### Statistical analysis

We summarized baseline participant characteristics across groups with AKI-SP1, AKI-SP2, no AKI on ICU admission and those without AKI on ICU admission who develop AKI during hospitalization with mean (SD) values for continuous variables and number and percentage for categorical variables. We used Cox proportional hazards regression to evaluate the association of AKI subgroups (AKI-SP1 and AKI-SP2) with incident RRT accounting for the competing risk of death. Additional methods can be found in the online supplement.

The RFU value for each aptamer-protein measurement in each sample was scaled by dividing it by the mean of all the aptamer-protein RFUs reported in that sample to account for urine sample dilution (mean normalized). Similar methods have been used to normalize urinary metabolomics data [31, 32]. Prior to this Somalogic also normalized the amount of protein loaded in their standard urine workflow. This produces an RFU abundance that is corrected in a similar manner to creatinine adjustment of urine biomarkers. The log_2_ transformation of mean normalized RFU values was used in regression which produces a regression beta estimate for the independent categorial variable that is the log_2_ fold change of the protein between comparison groups adjusted for age, sex, BMI and COVID-19 status. Significance was assessed at an FDR < 0.05.

To complete a pathway analysis, we used gene term enrichment over-representation analysis implemented in WebGestalt (webgestalt.org) on the 312 significant proteins that were different between AKI sub-phenotypes. We tested the proteins which were significantly up regulated in AKI-SP1 separate from those upregulated in AKI-SP2 to characterize the two phenotypes’ pathways independently.

To develop a urinary proteomic classification model which identifies patients with AKI-SP2, we made 1000 bootstrapped 75% training and 25% test splits of the data where the balanced random sampling was performed within the respective groups being classified (i.e. AKI-SP1 and AKI-SP2). The training sets were prescreened for the candidate proteins using the same regression methods as reported above to identify the proteins differentially abundant and selected those proteins with an FDR < 0.2. We then used the glmnet (v4.1–4) R package least absolute shrinkage and selection operator (LASSO) tenfold cross validation regression implementation to perform feature selection among this reduced set. We performed this for 1000 bootstrapped iterations with random splits to obtain average area under the curve (AUC) prediction classification performance and confidence intervals of the test sets across these iterations.

## Results

### Description of cohort

Among 173 ICU patients with sepsis from a suspected respiratory infection, 87 had no AKI, 66 had AKI-SP1 and 20 had AKI-SP2 on study enrollment (Table 1). Among patients without AKI on study enrollment, 38 (44%) subsequently developed AKI on average 8 (SD ± 9) days after ICU presentation. Overall, the mean (SD) age was 53 (16) years old, 61% were men, and 57% identified as White, 17% as Black, 12% as Asian and 21% as LatinX. Compared to patients with AKI-SP1, patients with AKI-SP2 had lower rates of COVID-19 (35% vs 68%), lower rates of diabetes mellitus (30% vs 38%) and higher rates of CKD (30% vs 14%). Table S1 provides a summary of the sTNFR1, Ang-1 and Ang-2 biomarkers used to classify the participants into AKI-SP1 and AKI-SP2.

**Table 1:** Cohort characteristics

	Total(N = 173)	No AKI (N = 87)		AKI SP1(N = 66)	AKI SP2(N = 20)	*P-value* comparing AKI-SP1 to AKI-SP2
		No AKI during hospitalization (n = 49)	AKI developed after study enrollment (N = 38)			
Demographics						
Age, mean (± SD)	53 (± 16)	52 (± 16)	52 (± 13)	56 (± 18)	52 (± 16)	0.34
Male, N (%)	105 (61)	30 (61.2)	24 (63.2)	42 (63.6)	10 (50)	0.28
Body mass index, mean (± SD)	32 (± 11)	33 (± 12)	36 (± 12)	31 (± 10)	31 (± 9)	0.87
Race, N (%)						–
White	98 (57)	25 (51)	27 (71)	36 (55)	10 (50)	–
Asian	21 (12)	6 (12)	1 (3)	13 (20)	1 (5)	–
Black	29 (17)	12 (25)	3 (8)	10 (15)	4 (20)	–
Native american	9 (5)	2 (4)	4 (11)	0 (0)	3 (15)	–
Pacific islander	3 (2)	0 (0)	2 (5)	1 (2)	0 (0)	–
Other	2 (1)	2 (4)	0 (0)	0 (0)	0 (0)	–
Unknown	11 (6)	2 (4)	1 (3)	6 (9)	2 (10)	–
Latinx	36 (21)	9 (18)	13 (34)	13 (20)	1 (5)	–
Comorbidities, N (%)						
Hypertension	87 (50)	23 (47)	20 (53)	37 (56)	7 (35)	0.10
Chronic kidney disease	26 (15)	5 (10)	6 (16)	9 (14)	6 (30)	0.09
Diabetes mellitus	53 (31)	12 (25)	10 (26)	25 (38)	6 (30)	0.52
Etiology for ICU Admission, N (%)						
COVID-19 Infection	117 (68)	33 (67)	32 (84)	45 (68)	7 (35)	0.01
Non-COVID Pneumonia	15 (9)	4 (8)	0 (0)	9 (14)	2 (10)	–
Non-COVID ALI (ARDS, aspiration and contusion)	6 (4)	1 (2)	0 (0)	2 (3)	3 (15)	–
Exacerbation of chronic lung disease (asthma, COPD, bronchiectasis and ILD)	6 (4)	3 (6)	1 (3)	1 (2)	1 (5)	–
Cardiac dysfunction (CHF, MI, arrythmia, and cardiac arrest)	13 (8)	4 (8)	3 (8)	4 (6)	2 (10)	–
Nonpulmonary sepsis (NSTI, bacteremia, cholangitis, peritonitis)	6 (4)	1 (2)	0 (0)	1 (1.5)	4 (20)	–
Other	10 (6)	3 (6)	2 (5)	4 (6)	1 (5)	–
ICU Characteristics at study enrollment						
Receipt of invasive mechanical ventilation, n (%)	119 (70)	29 (60)	32 (84)	47 (72)	11 (55)	0.15
APACHE III Score, mean (± SD)	83 (± 29)	72.6 (± 29.8)	94.3 (± 30.4)	84 (± 24)	88 (± 26)	0.51
SOFA Score, mean (± SD)	8.9 (± 4.2)	6.9(± 4.1)	9.5 (± 3.9)	9.2 (± 3.4)	11.8 (± 4.8)	0.01
Clinical outcomes, N (%)						
Positive bacterial blood culture	8 (7.3)	1 (3.7)	0 (0)	1 (2.0)	6 (35)	< 0.01
Blood cultures completed	110 (64)	27 (55)	16 (42)	50 (75.8)	17 (85)	0.40
Renal Replacement Therapy	24 (13.9)	0 (0)	5 (13)	7 (10.6)	6 (30)	0.04
In-hospital mortality	60 (35.3)	11 (22)	22 (58)	20 (30.3)	8 (40)	0.41

^*^Denominator to obtain percentage is all the blood cultures completed

### Risk of clinical outcomes between AKI sub-phenotypes

Consistent with previous studies, we found that patients with AKI-SP2 had a higher risk of RRT than AKI-SP1. The proportion of patients receiving RRT during hospitalization was 10.6% among AKI-SP1 and 30% among AKI-SP2 (Table 1** and **Table S2**).** The Kaplan–Meier curve for risk of RRT demonstrates the majority of new RRT occurred in the first two weeks after ICU admission (Figure S1). We saw no significant difference in hospital mortality between AKI sub-phenotypes (Table S3).

### Urinary proteomic profiles between patients with AKI-SP1 and AKI-SP2

**Fig. 1:**
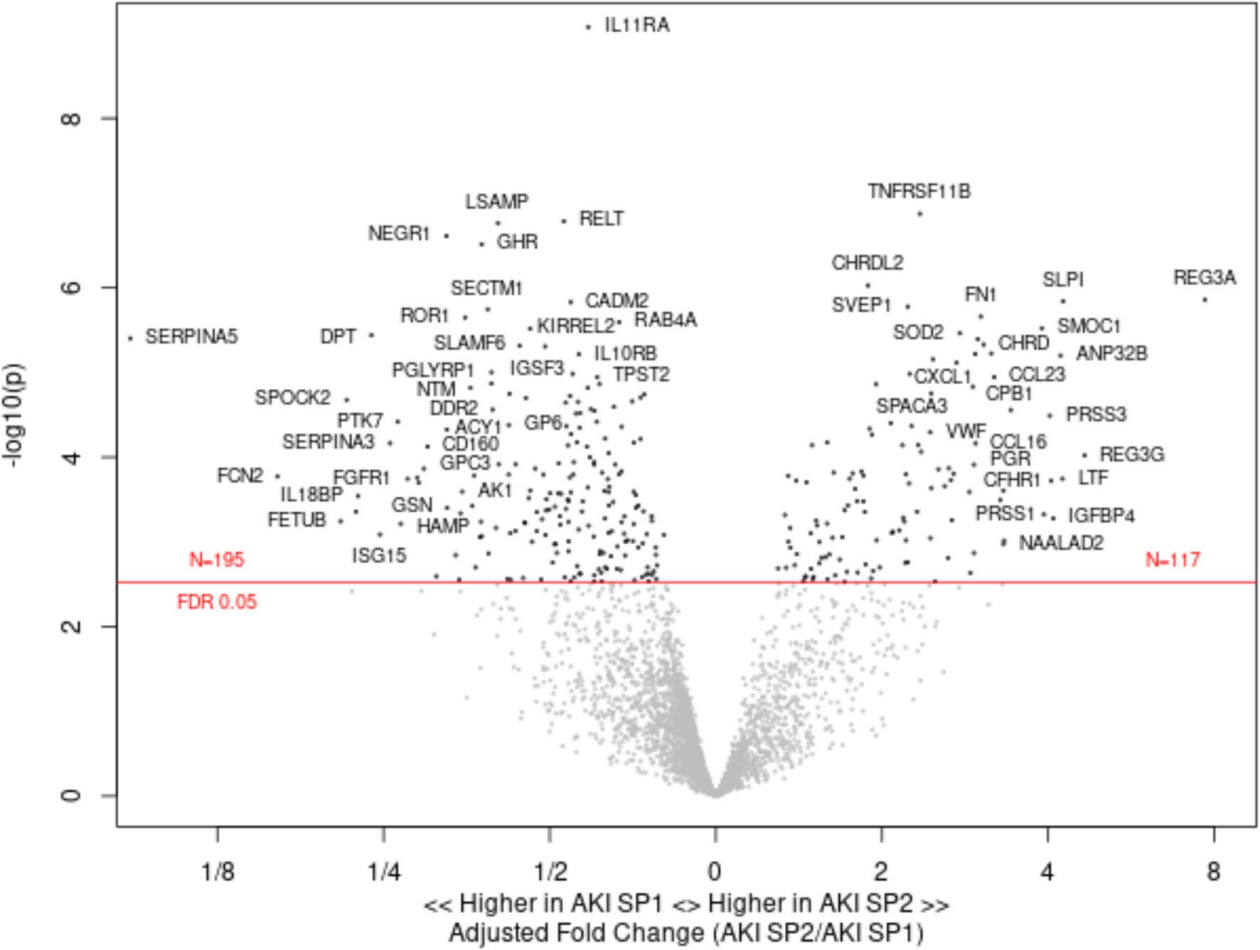
Comparison of urinary proteomic profile between patients with AKI-SP1 and AKI-SP2. Volcano plot showing 117 urinary proteins with higher abundance in AKI-SP2 and 195 urinary proteins with higher urinary abundance in AKI-SP1 adjusted for age, sex, COVID-19 diagnosis, and body mass index

Next, we sought to determine whether urinary proteins were different between these AKI sub-phenotypes. In total, 117 urine proteins were higher in AKI-SP2, while 195 urine proteins were higher in AKI-SP1 (*FDR* < *0.05*) (Fig. 1). In a sensitivity analysis, we compared the raw urinary protein RFUs between AKI sub-phenotypes. Using the raw urinary RFU values, we found a similar set of top proteins that were significantly different between AKI sub-phenotypes compared to the mean normalized RFU values for each protein (Figure S2). We also completed a sensitivity analysis adjusting for baseline CKD and found a similar set of top proteins between AKI sub-phenotypes (Figure S3).

Proteins involved in collagen deposition (GP6), podocyte derived (SPOCK2), proliferation of mesenchymal cells (IL11RA), and anti-inflammatory (IL10RB and TREM2) were among the proteins abundant in AKI-SP1. Urinary proteins involved in inflammation (TNFRSF11B), chemoattractant of neutrophils and monocytes (CXCL1 and REG3A) and oxidative stress (SOD2) were significantly associated with AKI-SP2. See Supplemental File 1 for summary statistics for each protein aptamer with each comparison.

Next, we compared the urinary proteomic profile between all patients without AKI on study enrollment (n = 88) and AKI-SP1 and found that no urinary proteins were significantly different (Figure S4). In contrast, patients with AKI-SP2 had significantly different urinary proteomic profiles compared to patients without AKI on study enrollment (Figure S5). In direct comparisons, we found that the log_2_ adjusted fold change in urinary proteins between AKI-SP2 vs no AKI and AKI-SP2 vs AKI-SP1 were highly correlated (Pearson's *r* = *0.91*, Figure S6). Moreover, a majority of the proteins were overlapping (n = 243) suggesting that AKI-SP1 and no AKI have a very similar urinary proteomic profile on study enrollment (Figure S7).

### Pathway analyses

**Fig. 2:**
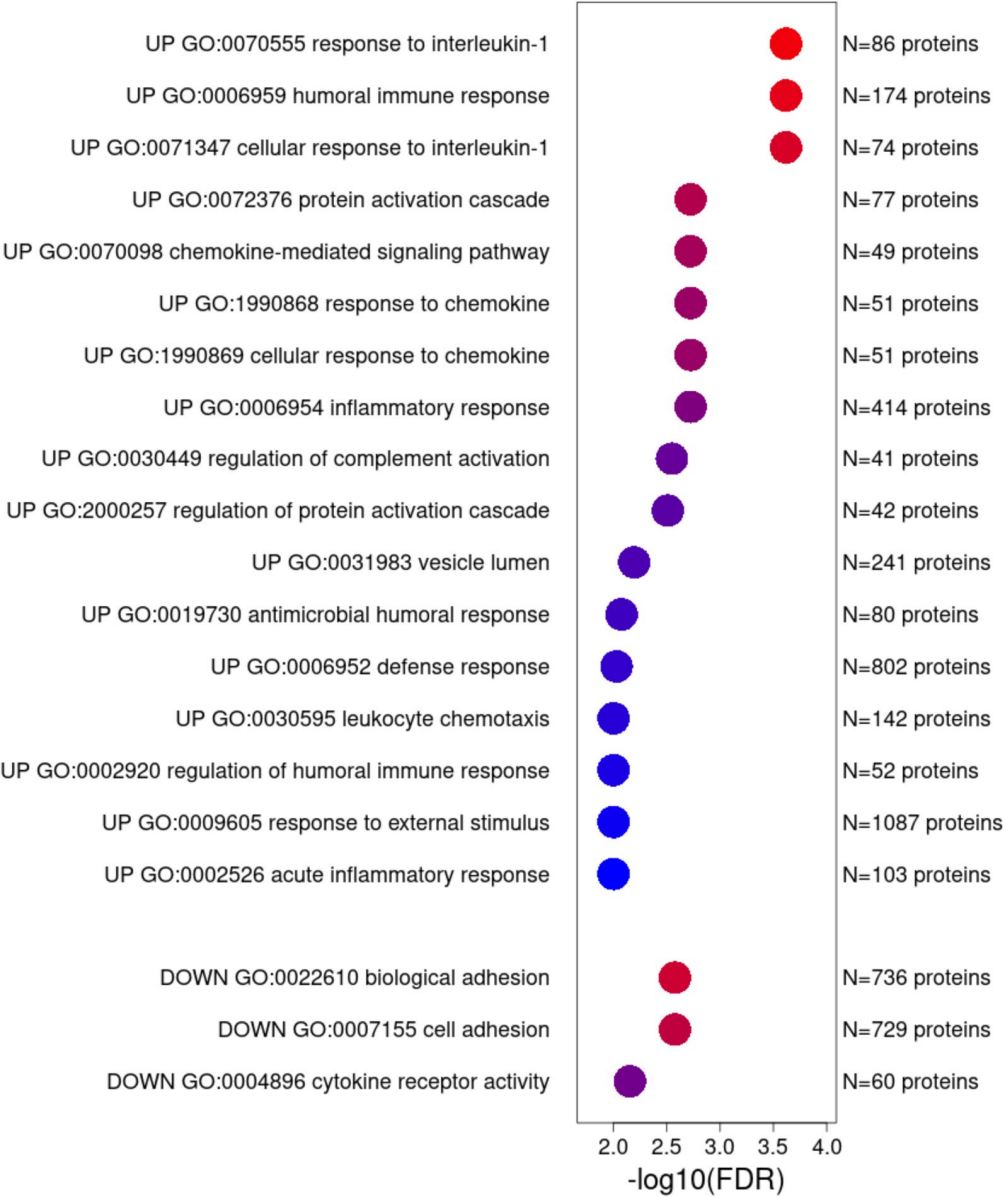
Pathway analysis of urinary proteins between AKI-SP1 and AKI-SP2. 17 pathways were upregulated in AKI-SP2, while 3 pathways were downregulated in AKI-SP2 using WebGestalt based on a FDR ≤ 0.1. To the left of the figure includes each pathway ID and to the right of the figure includes the total number of proteins within the pathway

We completed pathway analyses to annotate urinary proteins with differential abundance between AKI-SP1 and AKI-SP2. In WebGestalt pathway analysis, the gene-protein name term enrichment showed 17 pathways were significant for the proteins upregulated in AKI-SP2, while 3 pathways were significant for the proteins upregulated in AKI-SP1. Among these were pathways related to immune response, complement activation and chemokine signaling in AKI-SP2 and pathways of cell and biological adhesion were enriched in AKI-SP1 (Fig. 2** and **Supplemental File 1).

### Association of urinary proteins with risk of RRT

**Fig. 3:**
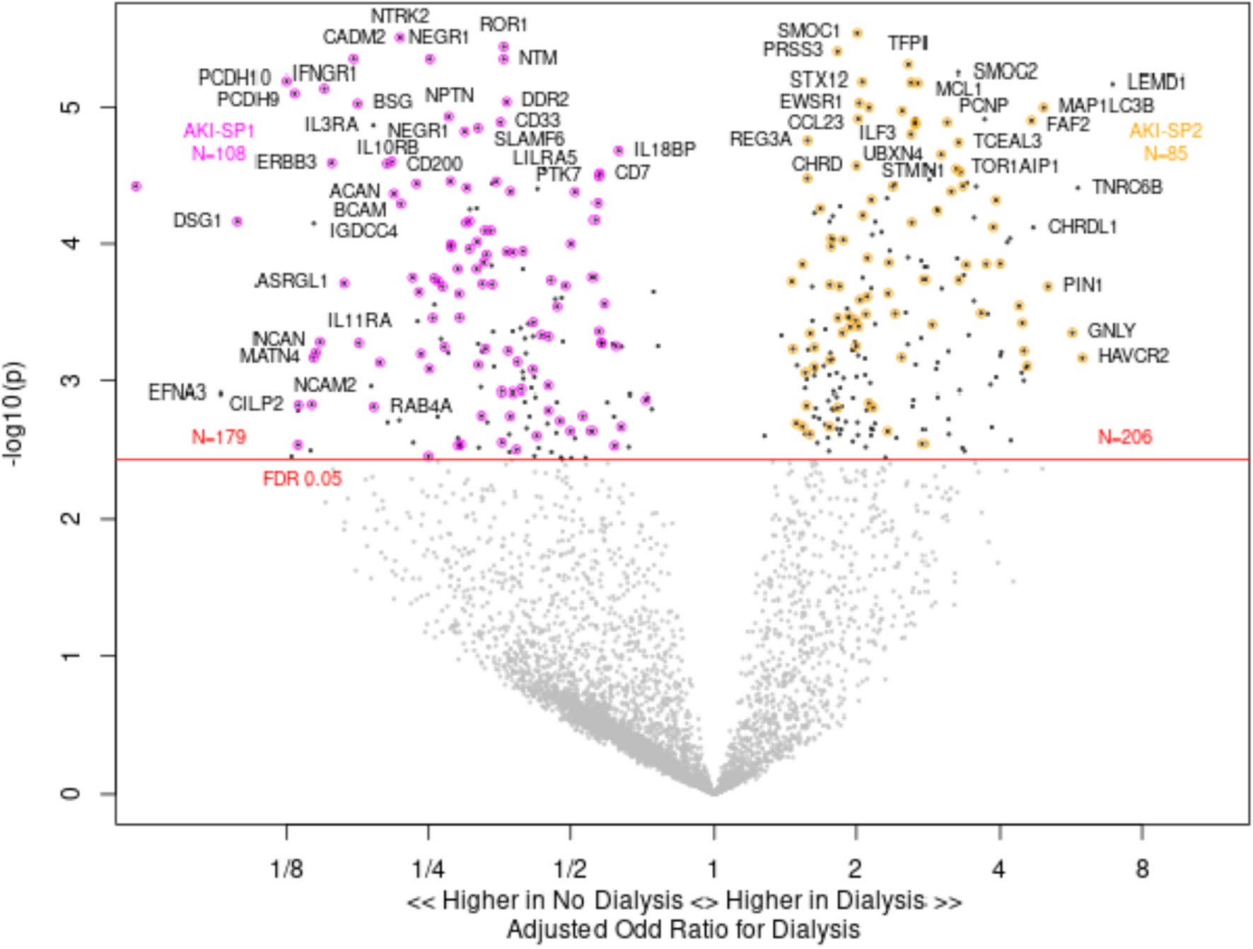
Association of urinary proteins with risk of RRT during hospitalization. Among 86 patients with AKI-SP1 or AKI-SP2, 13 (15%) developed RRT. A greater abundance of urinary proteins to the right of 1 (n = 206) demonstrate a higher odds of receiving dialysis and a greater abundance of urinary proteins to the left of 1 (n = 179) demonstrate a higher odds for not receiving dialysis. Urinary proteins that are also significantly associated with AKI-SP1 are colored purple and urinary proteins significantly associated with AKI-SP2 are colored gold to demonstrate the overlap in biological processes leading to AKI sub-phenotypes and also to risk of dialysis

Among 86 patients with either AKI-SP1 or AKI-SP2, 13 (15%) developed RRT during hospitalization. In total, greater abundance of 206 proteins in urine were associated with development of RRT, while a higher abundance of 179 urinary proteins were associated with a lower risk of RRT (*FDR* < *0.05*) (Supplemental File 1). We found high overlap with proteins that differentiated AKI sub-phenotypes and also were associated with risk of RRT. For example, of the 179 proteins that were associated with a lower risk of subsequent RRT, 108 of these proteins were also associated with risk of AKI-SP1. Similarly of the 206 urinary proteins that were associated with a greater risk of subsequent RRT, 85 of these proteins were associated with AKI-SP2. The overlap of proteins highlights the shared urinary protein biology between development of AKI sub-phenotypes and subsequent risk of RRT (Fig. 3).

### Rates of bacteremia in patients with AKI sub-phenotypes

With proteins of immune and complement activation and TLR expression increased in the urinary proteomic profile of patients with AKI-SP2, we sought to determine whether blood cultures positive for bacteria were more common in patients with AKI-SP2. We reviewed blood culture results in the first week of study enrollment and found that patients with AKI-SP2 were more likely to have detectable bacteria in their blood (35%) compared to patients with AKI-SP1 (2%) (*p* = *0.007*).

### Classification of AKI sub-phenotypes using the urinary proteome

To facilitate identification of AKI-SP2 on study enrollment through a non-invasive urinary sampling method, we developed urinary proteomic prediction models. We iteratively split the cohort into 1000 testing (25%) and training (75%) bootstrap sets within the classification groups and used LASSO regression to develop a urinary proteome prediction model for AKI-SP2 compared to patients without AKI and/or AKI-SP1 as well at AKI-SP1 versus AKI-SP2 and AKI-SP1 versus No AKI. We then combined the training and test sets for a final model with selected proteins for AKI-SP2 compared to patients without AKI and/or AKI-SP1. The bootstrap test datasets had a mean area under the curve (AUC) of 0.84 (95% CI: 0.66 – 0.98) to predict AKI-SP2 in comparison to no AKI and AKI-SP1 (Table 2**)**. The final LASSO model for AKI-SP2 versus AKI-SP1 and No AKI groups included 30 different urinary proteins (Supplemental File 1). Similar performance (AUC = 0.80 (95% CI: 0.56–0.99)) was seen when distinguishing AKI-SP2 from those participants with AKI-SP1. The difference between the two AUC bootstrap samplings was significant (two-sided t-test *p*-value < 2.2 × 10^–16^). We were not able to usefully predict classification differences between AKI-SP1 and No AKI (Table 2**)** which supports the observation in our regression analyses where we saw no proteins that were significantly different between No AKI and AKI-SP1.

**Table 2:** AUCs of urinary proteomic classification model for AKI-SP1, AKI-SP2 and no AKI among all patients

Group A			Group B			Classification performance	
	Train(N) 75%	Test(N) 25%		Train(N) 75%	Test(N) 25%	AUC	95% CI
AKI SP2	15	5	No AKI or AKI-SP1	114	39	0.84	0.66–0.98
AKI SP2	15	5	AKI SP1	49	17	0.80	0.56–0.99
AKI SP1	49	17	No AKI	65	22	0.60	0.48–0.72

Predicting the difference between Group A and Group B with 1000 bootstraps of test and train sets to estimate the average AUC and confidence interval. The final LASSO model for AKI-SP2 included 32 different urinary proteins

### Validation of aptamer specificity with ELISA measurements

We compared aptamer-based measurements with the corresponding Meso Scale Discovery immunoassay-based protein measurements of proteins found to be significantly associated with AKI sub-phenotypes (REG3A, MMP2, HAMP, RBP4, PRDX6) and candidate urinary biomarkers of kidney injury, such as KIM-1, NGAL, EGF, IL-18 and Ang-2 (Table S4). Prior to completing proteomics, Somalogic normalizes all urine samples to a similar total protein concentration by diluting the urine sample. We applied this dilution factor to the RFUs to calculate a neat (undiluted) value that is comparable to an immunoassay-based protein measurement. Among the five kidney injury biomarkers we found higher correlation for Ang-2 (Pearson’s r = 0.74) and KIM-1 (r = 0.6), moderate correlation for NGAL (r = 0.43) and no correlation for EGF (r = -0.04) and IL-18 (r = -0.01). Among the proteins associated with AKI sub-phenotypes we found higher correlation for REG3A (r = 0.86), moderate correlation for MMP2 (r = 0.46), HAMP (r = 0.42) and PRDX6 (r = 0.53). RBP4 was not correlated (r = 0.12). The combination of four biomarkers measured using an immunoassay (REG3A, MMP2, HAMP and PRDX6) with the clinical variables of age and sex had a AUC of 0.69 (0.5–0.93) to predict AKI-SP2 compared to AKI-SP1 and no AKI.

## Discussion

In sepsis-induced AKI, it has been particularly problematic to identify clinical subgroups with biologically distinct signatures. For example, clinicians have historically separated AKI into prerenal and acute tubular necrosis. However, multiple studies have shown poor reliability in identifying these two groups by clinicians [33] and enriching for acute tubular necrosis in AKI clinical trials has yet to show a benefit [34, 35]. In our previous work, we developed a validated 3-variable molecular classifier to identify two AKI sub-phenotypes. Here we demonstrate through measurement of 5,000 proteins on urine collected within 24 h of ICU admission that these two AKI sub-phenotypes each have distinct urinary profiles. Patients with AKI-SP1 were characterized by a reparative, regenerative phenotype and AKI-SP2 being characterized by an immune activation and inflammatory phenotype. We also demonstrate shared urinary protein biology between AKI sub-phenotypes and subsequent risk of RRT and highlight potential future therapeutic targets.

Relatively few studies have evaluated the urine proteome in ICU patients with sepsis-induced AKI [36]. The largest previous study included twelve patients with early recovery of kidney function matched to 12 patients with late/non recovery. Mass spectrometry was completed on urine samples and identified 8 differential proteins [37]. Among the 8 proteins, higher urinary concentrations of neutrophil gelatinase-associated lipocalin (NGAL) were associated with greater risk of late or never recovery of renal function. In our work, we also found that higher urinary NGAL was associated with AKI-SP2 (*FDR* = *0.035*) and eventual risk of RRT (*FDR* = *0.003*). The shared findings demonstrate the external generalizability of our cohort, and our work adds to previous urinary proteomic analyses by including a larger sample size of patients with AKI, measurement of 5,000 proteins and leveraging identification of AKI sub-phenotypes.

The identification of distinct urinary proteomic profiles between AKI sub-phenotypes may inform future testing of successful pre-clinical therapeutics tailored to underlying biology. For example, among the top proteins most abundant in AKI-SP1 was IL11RA. IL11 and IL11RA have been shown to be crucial to the development of fibrosis in AKI and CKD models, and anti-IL11 treatment as well as knockout of the IL11RA gene are protective [38, 39]. Another example is that a number of pro-inflammatory cytokines and pathways of inflammation were upregulated in patients with AKI-SP2. Thus, trials of anti-inflammatory therapies in AKI may be mixed [40–43] partly due to the inclusion of AKI-SP1 patients with different pathophysiology than AKI-SP1. In addition, urine chordin-like 2 (CHRDL2) was increased in AKI-SP2 and higher urine CHRDL2 was associated with risk of RRT. The chordin proteins are antagonists to bone morphogenetic /transforming growth factor-beta (BMP/TGF-beta) signaling and are a critical mediator to renal fibrosis, inflammation and apoptosis after kidney injury [44]. Preclinical studies have shown that modulation of BMP-7 prevents kidney fibrosis and improves survival in rodent kidney ischemia [45, 46] but application to clinical AKI has been less promising [47]. Our urine proteomic findings suggest that including all types of patients with AKI with diverse biology in therapeutic studies may prevent translation of promising pre-clinical signals to clinical AKI.

The ideal method to model protein measurements in a spot urine sample is debatable [48, 49]. One method is to index (i.e., divide) protein measurements by urine creatinine (UCr) concentrations. However, UCr concentration decreases as glomerular filtration rate falls and thus the proteins indexed to UCr may overestimate or underestimate the true association of these proteins with clinical outcomes and empirical data has supported this [48, 49]. For this reason, we normalized urine aptamer protein RFUs to the mean of the total RFUs of all proteins within the sample. In a patient with concentrated urine, we would expect that all the protein RFUs to be increased and in a patient with dilute urine for all the protein RFUs to decrease. Thus, normalizing to the mean of the total RFUs will maintain the relative difference among protein RFUs within sample but also account for urine dilution. This approach has been used to normalize urine metabolomics data [31, 32]. In a set of sensitivity analyses, we also present analyses using the raw urine protein-aptamer RFU and demonstrate high overlap in proteins between methods.

The strengths of our study include the use of a multiplex urinary proteomics platform on urine collected early after hospitalization to understand molecular signatures of AKI early after injury that are potentially modifiable. We also leverage the identification of AKI sub-phenotypes to improve the ability to identify differences in urinary proteomics. Another strength is the generalizability of our findings. In our analyses, we were able to replicate previously known protein-outcome associations, such as NGAL and risk of RRT. We also were able to leverage the large sample size to identify several novel proteins associated with AKI sub-phenotypes and risk of RRT.

Our study has several limitations. First, urine output was not used to identify patients with AKI because of missingness in urine output data with early enrollment after ICU admission. The absence of urine output might have selected patients with potentially higher AKI severity. Second, the AKI-SP2 population is small and demonstrated notable clinical differences from AKI-SP1 and given the population size we were unable to account for these differences. Third, we did not have access to an external validation cohort with urinary proteomic data available. Future directions will seek to test the difference in urinary proteomics in diverse sepsis-induced AKI populations. Fourth, aptamer-based proteomic methods may be affected by probe cross-reactivity and nonspecific binding. Moreover, few published datasets are available presenting the correlation of urine proteomic data with immunoassay measurements, which we show is correlated in seven out of 10 proteins evaluated. Fifth, patients who had severe AKI and were anuric on study enrollment could not contribute urine and thus urine proteomics would not be able to understand AKI pathology in this population.

In summary, among a population of patients admitted to the ICU with sepsis, we reproduce two AKI sub-phenotypes originally derived using plasma biomarkers, demonstrate distinct urinary proteomic signatures between AKI sub-phenotypes and highlight the shared urinary protein biology between AKI sub-phenotypes and risk of RRT. We also found that AKI-SP1 is not significantly different than patients without AKI on study enrollment when analyzing the urinary proteome. We highlight several key biological pathways in human AKI with corresponding pre-clinical studies demonstrating a future path for targeted therapeutics. We show that AKI-SP2 is associated with blood bacteremia. Finally, we show that AKI-SP2 can be classified using prediction modeling from urinary protein abundances. A deeper understanding of the human pathophysiology in sepsis-induced AKI may allow tailoring the study of potential therapeutics to AKI sub-phenotypes.

## Supplementary Information

Additional file 1.

## Data Availability

The datasets generated during and/or analyzed during the current study are not publicly available due to currently ongoing research studies, but the data are available from the corresponding author on reasonable request.

## Abbreviations

AKI: Acute kidney injury
AUC: Area under curve
CKD: Chronic kidney disease
ESKD: End-stage kidney disease
FDR: False discovery rate
GBJ: Generalized berk-jones
GO: Gene ontology
ICU: Intensive care unit
RRT: Renal replacement therapy
RFU: Relative fluorescent units
SCr: Serum creatinine
UCr: Urinary creatinine
