# Supplementary material for: Urinary proteomics identifies distinct immunological profiles of sepsis associated AKI sub-phenotypes
Source: Crit Care. 2024 Dec 18;28:419. doi: 10.1186/s13054-024-05202-9 (PMC11654061; doi:10.1186/s13054-024-05202-9)

**Online Supplement**

**Title:** Immune activation urinary proteomic profiles differentiate sepsis-associated acute kidney injury (AKI) sub-phenotypes

**Authors**: Ian B. Stanaway, PhD; Eric D. Morrell, MD; Linzee Mabrey, MD.; Neha A. Sathe, MD. MSc.; Zoie Bailey BS; Sarah Speckmaier; Jordan Lo BS; Leila R. Zelnick, PhD; Carmen Mikacenic, MD; Laura Evans, MD; Mark M. Wurfel, MD. PhD; Pavan K. Bhatraju, MD. MSc.

**Table of Contents**:

**eMethods**

**Table S1.** Plasma Biomarkers between patients without AKI and AKI sub-phenotypes

**Table S2.** Fine-Gray Risk of RRT by AKI sub-phenotypes compared to no AKI

**Table S3.** Risk of Hospital Mortality by AKI sub-phenotypes compared to no AKI

**Table S4.** Pearson’s correlations between the Urine Somalogic and the MSD measurements.

**Figure S1.** Kaplan-Meier curve of time to dialysis for AKI-SP1 versus AKI-SP2.

**Figure S2.** Comparison of urinary proteomic profile between patients with AKI-SP1 and AKI-SP2 using the raw urinary protein values.

**Figure S3**. Comparing of urinary proteomic profiles between AKI sub-phenotypes adjusting for baseline CKD.

**Figure S4.** Comparison of urinary proteomic profile between patients with AKI-SP1 and no AKI.

**Figure S5.** Comparison of urinary proteomic profile between patients with AKI-SP2 and no AKI.

**Figure S6.** Pearson's correlation comparison of the adjusted fold changes between the urinary protein analyses of AKI SP2/SP1 and AKI SP2/no AKI.

**Figure S7.** Overlap between urinary proteomics between two comparisons, 1) AKI-SP1 vs AKI-SP2 and 2) no AKI vs AKI-SP2.

**eMethods**

**Cox proportional hazards regression models**

We completed a series of *a priori* nested models controlling for potential confounding factors: age, sex, body mass index (BMI), COVID-19, diabetes mellitus, hypertension, and chronic kidney disease. We tested the proportional hazards regression assumption and found no statistically significant violation. We also tested the association of AKI sub-phenotypes with positive bacterial cultures in blood (bacteremia) in the first week after study enrollment using logistic regression with the R package glm() function while adjusting for the same covariates.

**Table S1****.** Plasma Biomarkers between patients without AKI and AKI sub-phenotypes

|  | **No AKI**  **(N=87)** | **AKI-SP1**  **(N=66)** | **AKI-SP2**  **(N=20)** | **Total**  **(N=173)** |
| --- | --- | --- | --- | --- |
| sTNFR1, pg/mL, Mean (SD) | 4359 (3871) | 4724 (3140) | 13867 (9435) | 5634 (5521) |
| Ang-1, pg/mL, Mean (SD) | 7566 (9193) | 3905 (3414) | 1599 (2553) | 5418 (7155) |
| Ang-2, pg/mL Mean (SD) | 6530 (8429) | 6833 (6675) | 34941 (30724) | 10011 (15576) |

**Table S2.** Fine-Gray Risk of RRT by AKI sub-phenotypes

|  | **No. at Risk** | **Events,**  **n (%)** | **Unadjusted: sHR**  **(95% CI)** | **p-value** | **Model 1: sHR**  **95% CI)** | **p-value** | **Model 2: sHR**  **(95% CI)** | **p-value** |
| --- | --- | --- | --- | --- | --- | --- | --- | --- |
| AKI-SP1 | 66 | 7 (10.6) | Ref | - | Ref | - | Ref | - |
| AKI-SP2 | 20 | 6 (30) | 3.77 (1.32-10.76) | 0.013 | 7.15 (2.68-19.10) | 8.62x10^-5^ | 41.83 (6.92-252.76) | 4.74x10^-5^ |
| Urinary Biomarker Prediction of AKI-SP1* | 68 | 7 (10.3) | Ref | - | Ref | - | Ref | - |
| Urinary Biomarker Prediction of AKI-SP2* | 18 | 6 (33.3) | 4.42 (1.54-12.68) | 0.0057 | 10.87 (3.48-33.95) | 8.6x10-5 | 39.95 (7.24-220.55) | 2.34x10-5 |

*30 urinary protein prediction model used to define AKI sub-phenotypes

Sub-distribution hazard ratio (sHR) models with the competing risk of death are provided. Model 1 adjusted for age, sex, body mass index, COVID-19. Model 2 adjusted for model 1 covariates and diabetes mellitus, hypertension, chronic kidney disease

**Table S3.** Risk of Hospital Mortality by AKI sub-phenotypes compared to no AKI

|  | No. at Risk | Events,  n (%) | Unadjusted HR  (95% CI) | p-value | Model 1: HR  95% CI) | p-value | Model 2: HR  (95% CI) | p-value |
| --- | --- | --- | --- | --- | --- | --- | --- | --- |
| AKI-SP1 | 66 | 20 (30.3) | Ref | - | Ref | - | Ref | - |
| AKI-SP2 | 20 | 8 (40) | 1.48 (0.65-3.38) | 0.35 | 1.40 (0.52-3.77) | 0.50 | 5.93 (0.91-38.75) | 0.063 |
| Urinary Biomarker Prediction of AKI-SP1* | 68 | 21 (30.9) | Ref | - | Ref | - | Ref | - |
| Urinary Biomarker Prediction of AKI-SP2* | 18 | 7 (39) | 1.32 (0.56-3.10) | 0.53 | 1.13 (0.40-3.16) | 0.82 | 2.94 (0.50-17.48) | 0.24 |

*30 urinary protein prediction model used to define AKI sub-phenotypes

Model 1 adjusted for age, sex, body mass index, and COVID-19. Model 2 adjusted for model 1 covariates and diabetes mellitus, hypertension, chronic kidney disease

**Table S4.** Pearson’s correlations between the Urine Somalogic and the MSD measurements.

| **Urine Biomarker** | **Number of Samples** | **Pearson’s Correlation Between Urine Aptamer and MSD** | **p-value** |
| --- | --- | --- | --- |
| Ang-2* | 100 | 0.74 | 2.3x10^-18^ |
| NGAL (LCN2) | 173 | 0.43 | 2.4x10^-9^ |
| KIM1 (HAVCR1) | 159 | 0.6 | 5.9x10^-17^ |
| IL18 | 169 | -0.01 | 0.91 |
| EGF | 153 | -0.04 | 0.59 |
| REG3A | 173 | 0.86 | 5.3x10^-51^ |
| MMP2 | 173 | 0.46 | 1.6x10^-10^ |
| HAMP (Hepcidin) | 173 | 0.42 | 5.7x10^-8^ |
| RBP4 | 173 | 0.12 | 0.13 |
| PRDX6 | 173 | 0.53 | 5.2x10^-14^ |

*73 urine samples had undetectable ang-2 concentrations using the Meso Scale Diagnostics immunoassay.

**Figure S1.** Kaplan-Meier time to dialysis for AKI-SP1 versus AKI-SP2.


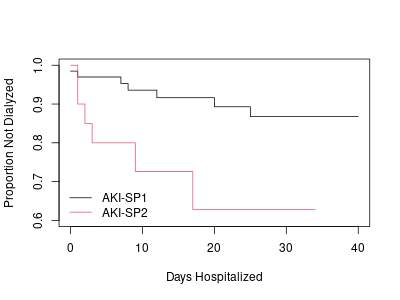


**Figure S2.** Comparison of urinary proteomic profile between patients with AKI-SP1 and AKI-SP2 using the raw urinary protein values. Volcano plot showing 93 urinary proteins with higher abundance in AKI-SP2 and 64 urinary proteins with higher urinary abundance in AKI-SP1 adjusted for age, sex, COVID-19 diagnosis, and body mass index.


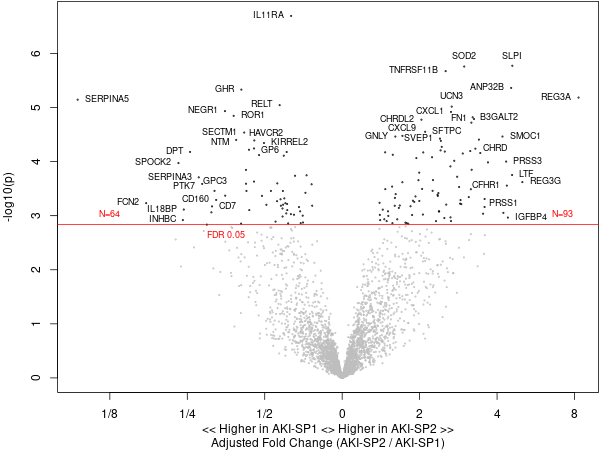


**Figure S3**. Comparison of urinary proteomic profiles between AKI sub-phenotypes adjusting for baseline CKD.


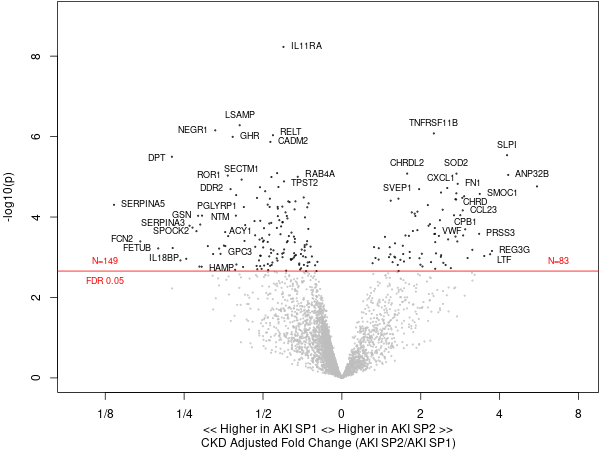


Volcano plot showing 83 urinary proteins with higher abundance in AKI-SP2 and 149 urinary proteins with higher urinary abundance in AKI-SP1 adjusted for age, sex, COVID-19 diagnosis, CKD and body mass index.

**Figure S4.** Comparison of urinary proteomic profile between patients with AKI-SP1 and no AKI.

**
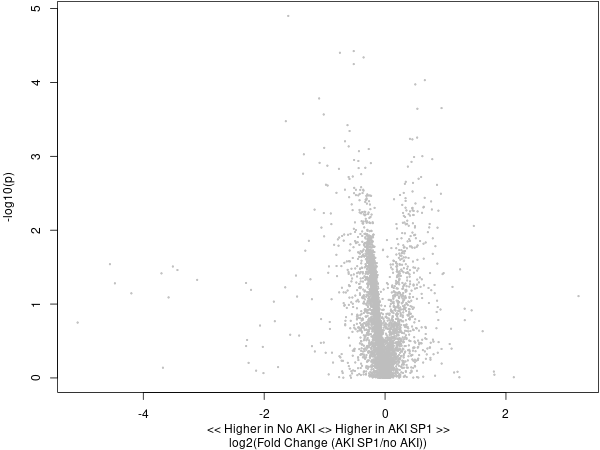
**

**Legend**. Volcano plot demonstrating no urinary proteins that are significantly different between AKI-SP1 versus no AKI.

**Figure S5.** Comparison of urinary proteomic profile between patients with AKI-SP2 and no AKI.


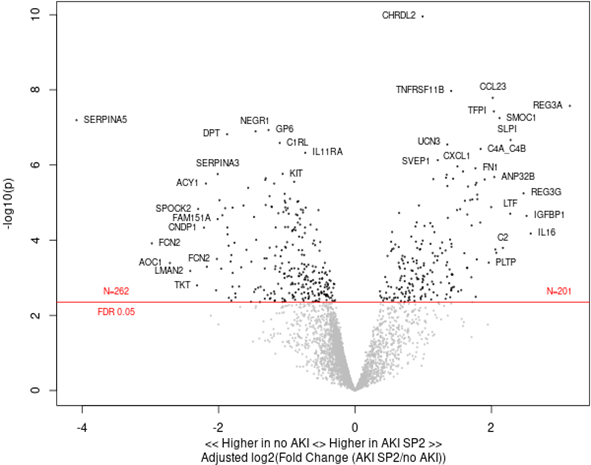


Volcano plot showing 201 urinary proteins with higher abundance in AKI-SP2 and 262 urinary proteins with higher urinary abundance in no AKI adjusted for age, sex, COVID-19 and body mass index.

**Figure S6.** Pearson's correlation comparison of the adjusted fold changes between the urinary protein analyses of AKI SP2/SP1 and AKI SP2/no AKI.


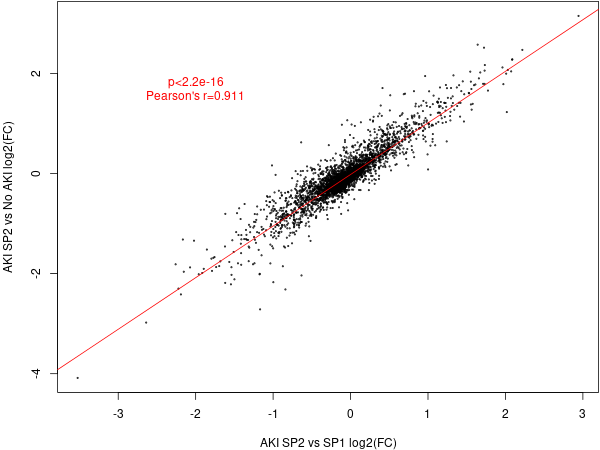


**Figure S7.** Overlap between significant (FDR<0.05) urinary proteomics between two comparisons, 1) AKI-SP1 vs AKI-SP2 and 2) no AKI vs AKI-SP2.


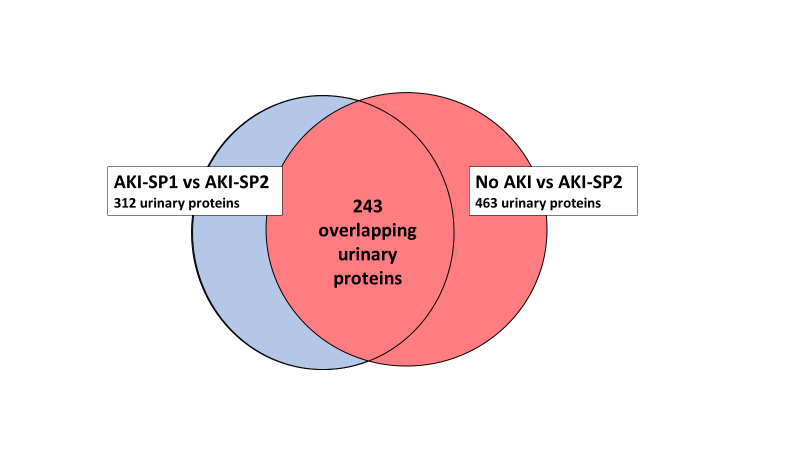

Supplement: Supplementary file 1 — Additional file 1. [file 13054_2024_5202_MOESM1_ESM.docx]
